# Detecting environmental barriers affecting older adult pedestrians via Gramian angular field-based CNN of smartphone sensor data

**DOI:** 10.3389/fpubh.2025.1697589

**Published:** 2025-11-05

**Authors:** Sungkook Hong, Hyunsoo Kim

**Affiliations:** Department of Architectural Engineering, Dankook University, Yongin, Republic of Korea

**Keywords:** Gramian Angular Field (GAF), convolutional neural network (CNN), older adult gait analysis, environmental barriers, wearable sensor walkability monitoring

## Abstract

**Introduction:**

Promoting safe walking among older adults requires precise identification of environmental barriers that disrupt gait. Traditional adult- and survey-based walkability assessments are labor-intensive and often miss transient hazards, while prior wearable-sensor methods—threshold-based acceleration, Maximum Lyapunov Exponent (MaxLE, a gait-stability index quantifying the local divergence of gait dynamics), and information entropy—either lack individual sensitivity or depend on aggregated data. This study introduces a framework that converts smartphone IMU time-series into Gramian Angular Field (GAF) images for classification by a lightweight CNN.

**Methods:**

Twenty older adults completed walking trials along a 1.2 km urban route featuring common barriers (uneven sidewalks, curb drops, narrow alleys, driveway crossings). IMU data were filtered, segmented into 2-s windows, transformed into 200 × 200-pixel GAF images, and evaluated under leave-one-subject-out cross-validation.

**Results:**

Among three benchmarks—peak-acceleration threshold, MaxLE (82.3% accuracy, F1-score = 0.45), and multi-user entropy—the GAF-CNN achieved 90.8% accuracy, 93.0% sensitivity, and 88.1% specificity, significantly outperforming the baselines (75–85% accuracy). Spatial mapping confirmed close correspondence between detected anomalies and true barrier locations.

**Discussion:**

These findings demonstrate that image-based deep learning provides a practical and interpretable solution for real-time, personalized detection of environmental barriers, offering a scalable tool for data-driven walkability enhancement in age-friendly urban design.

## 1 Introduction

Population aging has made the mobility of older adults an urgent public health concern (1), as the ability to walk safely and independently is essential for maintaining both physical health and social participation in later life (2). Walking is the most common and accessible form of exercise for older adults, yet it heavily depends on the quality and safety of the surrounding built environment (3, 4). In neighborhoods where sidewalks are continuous, smooth, and well-maintained, older adults are more likely to walk regularly and sustain social connections, thereby supporting longer independent living (5, 6). Conversely, environmental barriers—features of the built environment that impede comfortable walking—can discourage walking and reduce older adults’ mobility (7, 8). Examples of such barriers include uneven or broken sidewalks (9–11), curbs without ramps (12), obstructions on pathways (2), or high-traffic crossings without safe signals (1, 2, 11). These environmental barriers discourage outdoor activity and can ultimately contribute to physical decline and social isolation. Hence, eliminating or mitigating these barriers is essential because they restrict an individual’s ability to navigate their community and thus undermine the health and wellbeing benefits of walking (7).

Conventional approaches to assess walkability and identify environmental barriers rely on observational audits (13) and self-reported surveys (14) conducted by experts and local governments (15, 16). While these methods provide valuable qualitative insights, they are inherently limited in scalability, objectivity, and temporal resolution. First, manual audits and surveys are time-consuming and costly, especially when covering large urban areas (17). Second, static surveys may miss transient or context-dependent barriers—for example, temporary obstacles (construction zones, illegally parked cars) or environmental conditions like wet leaves that a one-time audit might not capture (18). Third, because comfort and mobility are relative to individual abilities, a one-size-fits-all checklist may overlook barriers that uniquely affect older or frail pedestrians (19). For instance, a slight incline or shallow step might pose no issue to a young adult but could be a significant hazard for someone with limited balance (20). Consequently, existing audit- and survey-based assessments tend to offer static snapshots of walkability that overlook the micro-scale and transient nature of the hazards that older pedestrians face (21).

Advances in mobile and wearable sensing technologies provide a promising pathway to overcome these limitations (22). Smartphones and wearable devices equipped with inertial measurement units (IMUs) can continuously capture detailed motion data while individuals walk, effectively transforming pedestrians into real-time environmental sensors (23–25). Environmental barriers cause subtle perturbations in gait—such as temporary loss of stability, increased variability, or irregular step timing (26, 27)—that can be detected through accelerometer and gyroscope signals (1). Under stable walking conditions, gait patterns remain periodic and consistent (28), whereas exposure to a barrier leads to transient deviations from normal dynamics. Detecting such anomalies enables the inference of environmental hazards indirectly, through changes in the gait itself (18, 29). This concept—using gait responses as a proxy for environmental quality—offers a data-driven approach to characterize walkability at high spatial and temporal resolution. However, despite this promise, the existing literature remains fragmented and methodologically limited in achieving robust and individualized detection of environmental barriers in real-world settings.

The motivation of this study arises from these unresolved challenges. Previous sensing-based studies have often relied on manually tuned thresholds or handcrafted features derived from acceleration magnitude or frequency spectra, which are sensitive to individual differences and fail to generalize across diverse walking styles (30, 31). Other approaches aggregate gait responses across multiple users to identify population-level patterns, which limits single-user, real-time detection (32). Moreover, many prior experiments have been conducted under controlled conditions, resulting in limited validation on outdoor urban routes where surfaces and obstacles vary dynamically. Therefore, there is a pressing need for an analytical framework that can autonomously learn complex gait dynamics without prior feature selection and that can reliably detect brief, transient instability induced by environmental barriers during naturalistic walking.

To address this need, this study aims to develop a Convolutional Neural Networks (CNN)-based abnormal gait detection method using Gramian Angular Field (GAF) images of smartphone IMU data and evaluate its performance for identifying environmental barriers on pedestrian paths. In addition to this, the authors conduct a comparative analysis against three baseline techniques from prior work: (1) an acceleration SVM-based threshold method, (2) a Maximum Lyapunov Exponent (MaxLE)-based gait stability method, and (3) the Information Entropy method. The authors focus on older adult pedestrians (age ≥65) as the target population, given their high susceptibility to mobility barriers.

The contributions of this work are threefold. First, the authors introduce a novel application of GAF transformation in the context of urban walkability, demonstrating how time-series IMU data can be leveraged for image-based deep learning to detect micro-scale environmental hazards. Second, the authors present a comprehensive evaluation using real-world data collected from older walkers in a natural urban setting, showing that the proposed GAF-CNN approach achieves superior accuracy and robustness in barrier detection. Third, this paper demonstrates how gait-based anomaly detection using GAF-CNN can yield actionable insights for urban planning and age-friendly city initiatives. By showing that well-calibrated, segment-level EB scores align closely with real barrier locations, the study highlights a pathway for integrating wearable-sensor analytics into evidence-based barrier removal and infrastructure improvement strategies. Rather than remaining at the level of abstract walkability indices, the proposed approach enables fine-grained, spatially precise detection of obstacles that older pedestrians actually encounter. In doing so, it underscores the potential of combining time-series analysis, computer vision-inspired transformations, and urban health perspectives to operationalize smart, data-driven interventions for safer and more inclusive environments. The key contribution of this work lies in bridging the gap between subjective, labor-intensive field assessments and automated, personalized sensing analytics, offering a methodological foundation for real-time environmental barrier detection and age-friendly urban design.

## 2 Related works

A growing body of literature demonstrates the feasibility of wearable sensor-based barrier detection. Previous studies showed that defective sidewalk segments could be identified by analyzing irregularities in pedestrians’ acceleration patterns collected via a waist-mounted IMU (33–36). Other researchers linked environmental barriers with changes in quantitative gait stability metrics: for example, Bisadi et al. (37) observed that walking through disordered neighborhoods led to higher average MaxLE values (indicating more chaotic, less stable gait) and elevated heart rates in participants. In related work, Bisadi et al. (38) combined multiple wearable sensors – including electrodermal activity (EDA), blood volume pulse, and gait dynamics (MaxLE) – to evaluate neighborhood built environments, essentially measuring pedestrians’ stress and instability as they walked. Zeile et al. (39) took a broader approach by correlating biosensor data with geospatial analysis to define walkability, laying groundwork for using physiological responses (like heart rate or skin conductance) as proxies for environmental comfort. Across these studies, a recurring theme is that abnormal pedestrian responses tend to coincide with environmental stressors or hazards, confirming the fundamental premise that gait deviations can serve as a marker for barriers (30).

However, many early approaches relied on simple features of the acceleration signal, primarily the magnitude of motion (sometimes termed the signal vector magnitude, SVM) (40, 41). For instance, one might set a threshold on resultant acceleration and flag any segment exceeding that threshold as a “stumble” or abnormal event. While straightforward, intensity-based methods are prone to both false negatives and false positives (42). Some barriers elicit subtle gait changes rather than large spikes in acceleration – especially among cautious individuals who slow down for obstacles, producing only a mild change in SVM. These may be missed by a simple threshold tuned to detect larger events (31). Conversely, highly active or spontaneous movements (e.g., an older person increasing speed briefly or turning head suddenly) can momentarily raise acceleration magnitude and trigger false alarms in the absence of any external hazard (43). In fact, raw acceleration ranges vary significantly by individual due to differences in body size, gait style, and personal caution, making it difficult to choose a universal SVM threshold (30). MaxLE-based measures focus on gait stability and might better capture subtle instability; yet calculating Lyapunov exponents over short walking intervals can be noisy and requires careful parameter selection (embedding dimension, etc.), which may limit reliability on a single crossing of a barrier (32). Moreover, MaxLE and other chaos metrics often need relatively long, steady data windows for accuracy, whereas an obstacle encounter is a brief event. Information-entropy approaches (1) convert IMU data into location-specific response distributions and compute Shannon entropy to quantify environmental unpredictability; this design effectively reveals population-level “hotspots” but depends on multi-user aggregation, making single-user, real-time detection difficult. In summary, prior wearable strategies either (1) rely on hand-crafted features with ad-hoc thresholds that struggle to generalize across individuals, or (2) require pooling data across participants or time, sacrificing immediacy.

More recently, advances in machine learning have enabled the use of data-driven models to classify gait patterns without explicit feature engineering. Support Vector Machines (SVMs) trained on handcrafted gait descriptors improved classification accuracy for irregular surfaces, but their performance remains limited by the choice of features and inability to model long-term dependencies (40, 41). Deep learning architectures such as CNNs and Long Short-Term Memory (LSTM) networks have shown notable promise in capturing complex temporal structures in IMU signals (44). For example, Hwang et al. (45) demonstrated that an LSTM-based model could effectively classify abnormal gait related to joint impairment. Nevertheless, most deep learning studies have been confined to controlled laboratory conditions or clinical datasets, where variability in environmental context is minimal. Their applicability to real-world urban walking, where surface types and obstacles change dynamically, remains underexplored.

Collectively, these prior studies reveal three major research gaps. First, threshold-based and handcrafted-feature approaches lack robustness and generalizability across individuals. Second, population-aggregated entropy and stability metrics capture macro-level trends but cannot support personalized or real-time hazard detection. Third, while deep learning has shown strong potential, its validation on uncontrolled outdoor walking data from older adults remains limited, and empirical evidence under field conditions is scarce. To address these research gaps, this paper proposes a novel deep learning-based framework to detect environmental walking barriers by analyzing wearable sensor data, specifically using GAF visualization and a CNN classifier. Our approach is inspired by recent advances in human activity recognition, where data-driven models markedly improved recognition accuracy by learning complex patterns directly from sensor signals. GAF encoding transforms a time-series into an image that preserves temporal dynamics as spatial patterns (46). In a GAF, each element of the image represents the trigonometric relationship (e.g., cosine of sum) between two time points, effectively capturing the intrinsic correlations within the signal. By converting IMU time-series segments into GAF images, subtle differences in gait dynamics (frequency shifts, phase changes, irregular oscillations) become visually salient features that a CNN can learn. This approach is advantageous because it does not require pre-selecting which signal feature (peak, variance, entropy, etc.) is important – the CNN can autonomously infer the relevant patterns associated with abnormal gait (47). Furthermore, CNNs have proven highly successful in time-series classification when paired with such time-series-to-image encodings, often outperforming traditional feature-based methods. For example, Serenelli et al. (48) found that GAF encodings combined with deep CNN models achieved the highest accuracy in classifying physiological signals (stress vs. calm conditions) compared to other encoding methods, attributing the success to GAF’s ability to preserve temporal dependencies in a 2D format that CNNs handle well. Based on this study, the authors hypothesize that a GAF-CNN model can similarly capture the nuanced gait alterations caused by environmental barriers more effectively than conventional methods. This approach enables reliable identification of transient gait instability caused by environmental barriers and demonstrates feasibility under realistic urban conditions, thereby extending deep learning–based gait analysis from laboratory experiments to practical, city-scale walkability assessment for aging populations.

## 3 Methodology

### 3.1 Research framework

**Figure 1:**
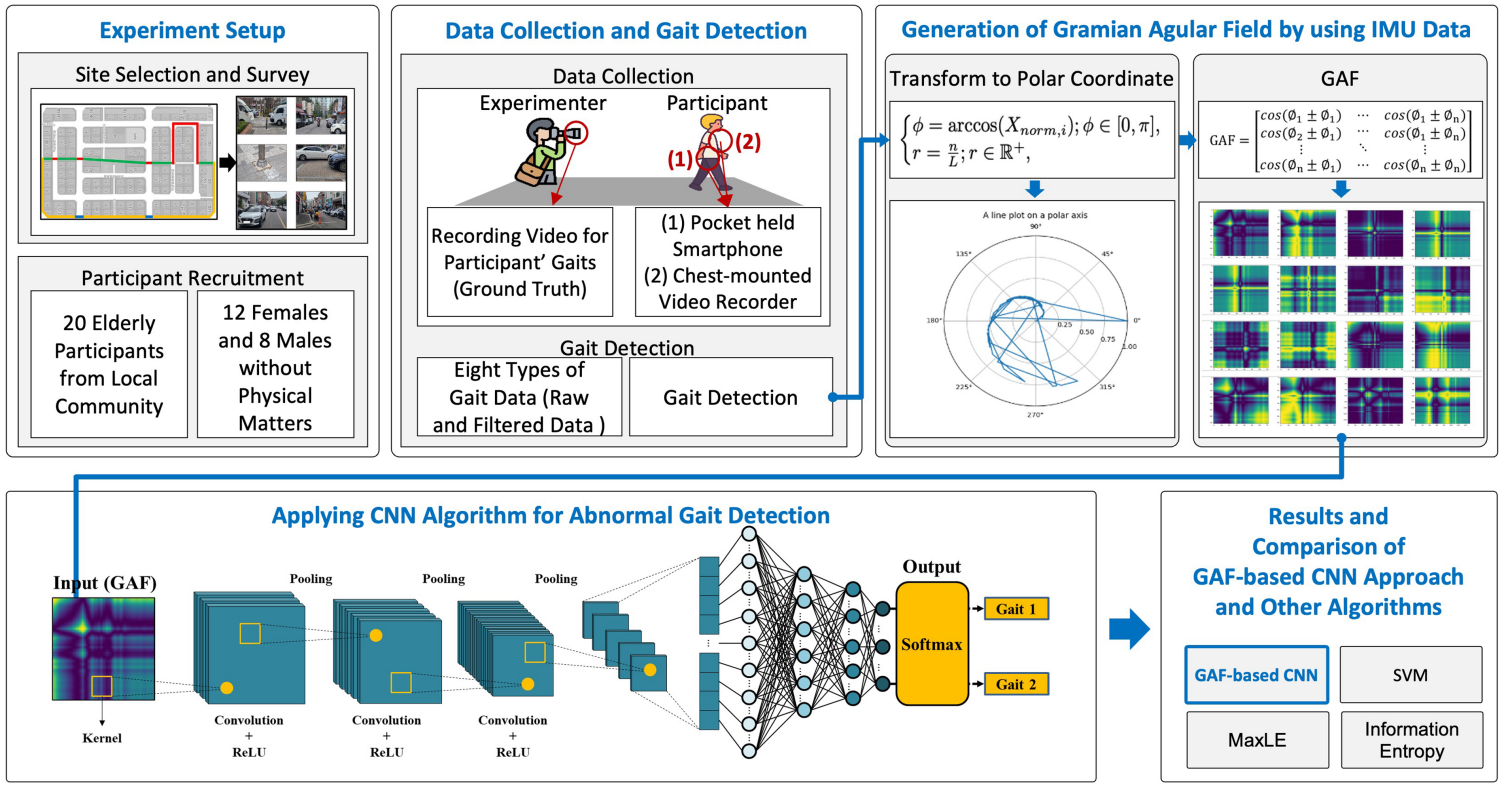
Research framework.

The overall research framework for identifying environmental barriers via older adults’ gait analysis is depicted in Figure 1. This multi-stage process begins with the Experiment Setup, where we carefully select and survey the study site and recruit participants. Specifically, a 1.2 km urban loop containing common pedestrian hazards (e.g., cracked sidewalks, curb drops, narrow alleys, driveway crossings) is surveyed and mapped. Twenty older adult volunteers (12 female, 8 male) without significant mobility impairments are recruited from the local community to ensure representative gait data under realistic conditions.

Once the site and participants are confirmed, the Data Collection and Gait Detection stage proceeds. Each participant completes two walking trials around the loop while carrying a smartphone in a pocket or mounted at the chest. Simultaneously, an experimenter records video footage to establish ground truth for each individual’s gait events (normal vs. abnormal). The smartphone’s built-in IMU (accelerometer and gyroscope) captures raw and filtered gait signals as the participant traverses both obstacle-free and barrier-laden segments. From these continuous sensor streams, distinct gait windows are detected and labeled according to whether they coincide with a known barrier location.

Next, in the Generation of GAF by Using IMU Data stage, each labeled gait segment is transformed into a two-dimensional image. The preprocessed accelerometer magnitude is first normalized and re-expressed in polar coordinates. A Gramian Angular Summation Field (GASF) matrix is then computed and produces a 200 × 200 pixel image that encodes pairwise angular relationships across the time-series. Representative GAF images for normal and abnormal gait illustrate how periodic walking yields a regular lattice pattern, whereas barrier-induced gait perturbations manifest as localized distortions.

The resulting GAF images serve as input to the CNN Algorithm for Abnormal Gait Detection. A lightweight convolutional neural network—comprising three convolutional-pooling blocks followed by a fully connected classifier and a softmax output—learns to distinguish normal versus abnormal GAF patterns. Each convolutional layer applies ReLU activations and max-pooling to hierarchically extract salient spatial features from the image representation of gait. The trained CNN ultimately outputs a probability distribution over two gait states (normal, and abnormal—severe perturbation), enabling fine-grained classification.

Finally, in the Results and Comparative Analysis stage, the GAF-CNN’s performance is benchmarked against three baseline algorithms: (1) an SVM using peak acceleration magnitude, (2) a MaxLE stability metric, and (3) an information entropy method. Classification metrics (accuracy, sensitivity, specificity, F1-score) and spatial correspondence between detected anomalies and true barrier locations are compared to demonstrate the proposed framework’s advantages. As shown in Figure 1, this end-to-end pipeline—spanning site selection to algorithmic comparison—provides a comprehensive methodology for real-time, personalized detection of environmental barriers based on older adults’ gait.

### 3.2 Experimental site and participants

**Figure 2:**
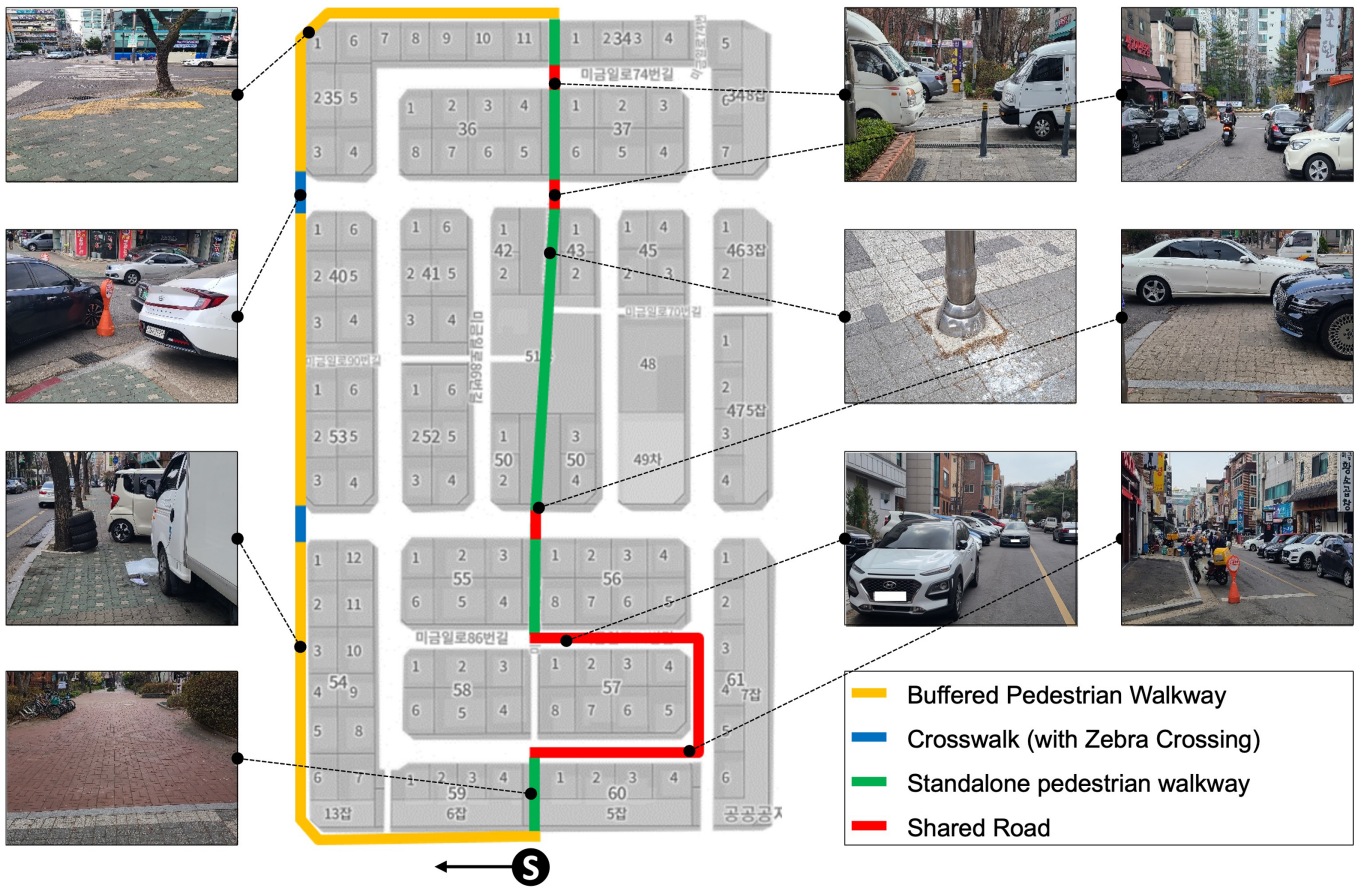
Experiment site overview.

This research was conducted in a busy urban neighborhood selected for its diverse pedestrian infrastructure and presence of common walking barriers. The study site is a 1.2 km loop in a city center, encompassing residential streets and commercial blocks. Along this route, we identified several representative environmental barriers frequently encountered by seniors: a section of uneven sidewalk with cracks and height mismatches, two intersections lacking curb ramps (requiring a step off the curb), a narrow alleyway (~0.8 m width) cluttered with bins, and segments of a mixed-use path where vehicles cross the sidewalk (driveway entries causing potential conflict). These features provided a varied testbed including both fixed obstacles (infrastructure-related) and more dynamic challenges (e.g., moving vehicles). The detailed route and features are illustrated in Figure 2.

The authors recruited 20 older adult volunteers (aged 65–80 years) from the local community. Participants were screened to ensure they were ambulatory without need of assistive devices and had no severe neurological or musculoskeletal disorders that would independently affect gait. Basic demographics of the participant group are summarized in Table 1. The sample (12 women and 8 men) had a mean age of 70.4 years (SD 4.1). All participants provided informed consent, and the experimental protocol was approved by the Institutional Review Board of the authors’ university (IRB No. 2025–04–015-022). To familiarize participants with the route and minimize anxiety, each individual was guided along the path once before data collection, with hazards pointed out so they could exercise appropriate caution.

**Table 1:** Characteristics of the older adult participants.

Characteristics	Value
Age (years), mean ± SD	70.4 ± 4.1
Female: Male	12: 8
Height (cm), mean ± SD	165.2 ± 7.5
Weight (kg), mean ± SD	68.9 ± 10.3

### 3.3 Data collection procedure

Each participant completed two walking trials around the designated loop at their normal, comfortable walking speed (resting as needed between trials). We used a standard Android smartphone (Samsung Galaxy S23) as the sensing device, exploiting its built-in IMU. The smartphone was secured to the participant’s body at the waist level, centered near the lower back using an elastic belt pouch (approximating the lumbar attachment used in prior gait studies) (34, 36). This placement was chosen as it provides a stable representation of whole-body motion and is less prone to extraneous arm movements than a pocket or handbag. The phone’s 3-axis accelerometer and 3-axis gyroscope were recorded at 100 Hz throughout each walk. Additionally, the phone’s GPS was logged at 1 Hz to later assist with mapping sensor data to spatial locations along the route.

Participants were instructed to walk naturally but to exercise caution at any perceived hazard. An investigator followed at a distance, noting the times or positions (via a handheld GPS tracker) when the participant traversed each predefined barrier location. These observations served as ground truth markers for analysis. It should be noted that not every participant was affected by each barrier in the same way—some navigated an obstacle smoothly, while others slowed down markedly or stumbled slightly. This inter-subject variability in responses is expected and indeed is the rationale for using sensitive detection methods beyond simple threshold triggers.

### 3.4 Data preprocessing and segmentation

Raw sensor data were exported from the smartphone and preprocessed in MATLAB and Python. First, we applied a low-pass Butterworth filter (4th order, cutoff 5 Hz) to the accelerometer and gyroscope signals to remove high-frequency noise and sensor jitter (49). This cutoff preserves the frequency content of normal gait (typically 1–2 Hz step frequency plus harmonics) while attenuating sudden spikes that do not reflect actual body motion (such as phone micro-vibrations). The filtered accelerometer readings were then resolved into a single composite magnitude: for each time sample, we computed the resultant acceleration as Equation 1.


(1) $$\text{Signal Vector Magnitude}(\mathrm{SVM})=\sqrt{{\left(x_{\mathrm{acc}}\right)}^{2}+{\left(y_{\mathrm{acc}}\right)}^{2}+{\left(z_{\mathrm{acc}}\right)}^{2}}$$

This signal vector magnitude collapses the 3-axis data into one dimension representing overall movement intensity, making the analysis orientation-invariant. Past research indicates that acceleration magnitude is a reliable measure of gait intensity and can capture events like trips or stumbles regardless of phone orientation (41).

Next, the continuous data stream for each trial was segmented into short windows for analysis. The author used a fixed sliding window of 2.0 s duration (with 50% overlap between consecutive windows). At the average walking speed of participants (~1.0–1.2 m/s), a 2-s window corresponds to roughly 2–3 stride cycles, which is short enough to localize an event (e.g., crossing a crack) yet long enough to capture a representative pattern of gait dynamics before, during, and after the event. Each window was labeled as either “normal” or “abnormal” gait based on whether it encompassed an environmental barrier encounter.

To assign labels, the author used the timestamped log of barrier locations from the observer notes: any window in which the participant was within ~ ± 1 m (spatially) of a known barrier point was labeled as an abnormal gait segment (since the act of negotiating the barrier occurs in that window). Windows occurring on straight, even sidewalk with no obstacles were labeled normal gait. By this method, we obtained a set of labeled segments for each participant’s trials. On average, each walk generated about 50–60 windows, of which ~10 were abnormal (depending on how many barriers the participant actually experienced; for example, if a participant walked very cautiously over a crack without any perturbation, that segment might not show any clear abnormality but was still labeled abnormal due to the presence of the barrier). To minimize ambiguity, we excluded a few borderline cases from training – e.g., if a participant stopped to talk (unrelated to a barrier) or if two barriers occurred in one short span (none did in our design). After segmentation and labeling, the data from all participants were aggregated, yielding a balanced dataset of 780 segments (400 normal vs. 380 abnormal).

### 3.5 Gramian Angular Field (GAF) image generation

**Figure 3:**
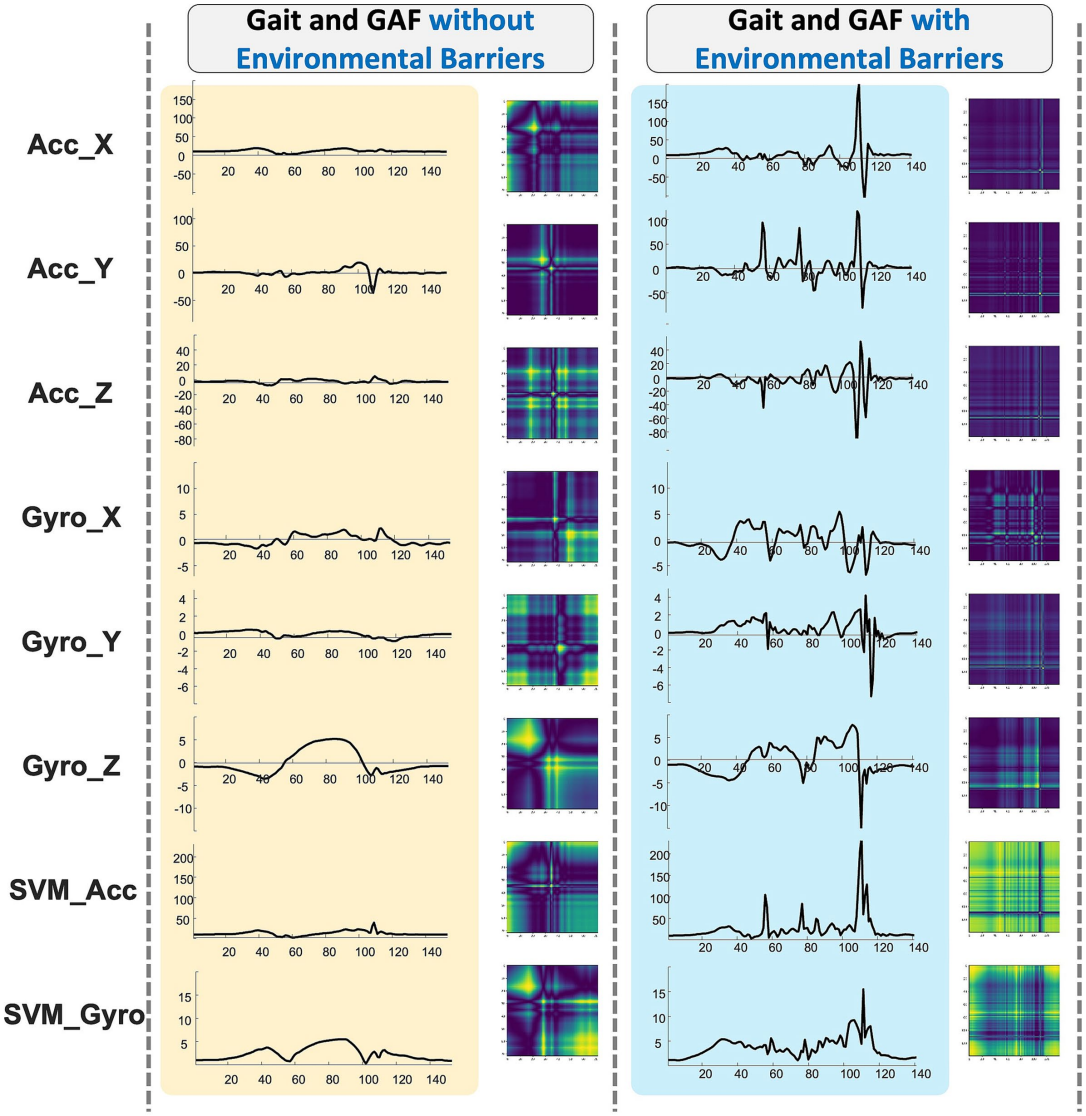
IMU time series and Gramian Angular Field (GAF) visualizations of older adults’ gait: comparison of segments without and with environmental barriers.

After filtering and windowing the smartphone IMU signals, each 2 s gait segment is converted into an image representation that preserves its temporal dynamics. Figure 3 contrasts the procedure for a segment traversed without an environmental barrier (left, yellow panel) and one with a barrier (right, blue panel). For every sensor channel—three axis acceleration (Acc_X, Acc_Y, Acc_Z), three-axis angular velocity (Gyro_X, Gyro_Y, Gyro_Z), and the derived signal-vector-magnitude curves (SVM_Acc, SVM_Gyro)—the raw values $x_{t}$ are first min-max normalized to [−1, 1]. Each normalized point is then mapped to polar coordinates as Equation 2.


(2) $$\theta_{t}=\arccos(x_{t}),r_{t}=\frac{t}{L}$$

where L is the window length. Forming all pairwise angular sums yields the Gramian Angular Summation Field as following Equation 3.


(3) $$\mathrm{GA}F_{\mathrm{ij}}=\cos(\theta_{i}+\theta_{j}),i,j\in [1,L]$$

which is rendered as a 200 × 200 pixel matrix. The eight channels are processed in parallel, producing a sequence of single-channel GAF images that can be later be combined for CNN input.

The left column of Figure 3 shows that normal, obstacle-free walking generates highly regular GAF lattices—periodic cross-hatch patterns reflecting consistent stride-to-stride correlations. In contrast, segments containing a barrier (right column) display pronounced distortions: phase shifts, amplitude spikes and irregular streaks appear in both the time-series plots and the corresponding GAF images. These visual disruptions capture subtle gait compensations such as hesitation, shuffling or a sudden jolt that may not be evident from a single scalar feature. Because the GAF encodes all temporal relationships within the window, it provides a rich 2-D texture that a CNN can exploit, enabling data-driven detection of abnormal gait events without hand-crafted thresholds.

### 3.6 CNN model architecture and training

The authors implemented a relatively lightweight yet sufficiently expressive convolutional neural network to classify each Gramian Angular Field (GAF) image as either normal or abnormal gait. A single-channel 200 × 200 GAF image enters the network and first passes through a convolutional layer with 16 filters of size 5 × 5 (stride = 1) followed by a ReLU activation and 2 × 2 max-pooling. The second convolutional block doubles the filter count to 32 with 3 × 3 kernels, again followed by ReLU and 2 × 2 pooling. A third convolutional layer, also with 32 filters of 3 × 3, applies ReLU activation but omits further pooling so that finer spatial detail is preserved. The resulting feature maps are flattened into a one-dimensional vector and fed to a fully connected layer containing 64 neurons with ReLU activation, after which a two-unit soft-max layer outputs the probabilities that the segment represents normal or abnormal gait. In total, the model comprises roughly 100,000 trainable parameters—large enough to learn nuanced patterns yet small enough for efficient training on our dataset. Coding was performed in Python with TensorFlow/Keras.

To enhance generalization, the author applied modest data augmentation: each training image was occasionally rotated by ±5 degrees or shifted a few pixels, simulating natural variability in phone orientation and walking speed. Optimization employed the Adam algorithm with a learning rate of 0.001 and binary cross-entropy loss; training ran for up to 50 epochs with early stopping after five epochs of stagnant validation loss. Generalizability was assessed via leave-one-subject-out cross-validation: for every fold the network was trained on data from 19 participants and tested on the remaining participant, ensuring that performance reflects subject-independent prediction. Hyperparameters such as filter size, depth and dense-layer width were tuned on an internal validation split through grid search. Finally, to quantify the benefit of the GAF representation, we also trained a comparable one-dimensional CNN that ingests the raw 200-sample acceleration sequence; its results are reported alongside the proposed model in Section 3 (Results).

#### 3.6.1 Hyperparameter tuning and model selection

To make model selection explicit and reproducible, we adopted a nested procedure within each leave-one-subject-out (LOSO) fold. The 19-subject training split was further partitioned into an inner validation set (subject-stratified, 20% of training windows) for hyperparameter search. We performed a coarse-to-fine grid search over the ranges in Table 2. The primary selection criterion was window-level AUC-PR (given class imbalance), with ECE (Expected Calibration Error) as a secondary tie-breaker to favor better-calibrated models. The finally selected configuration was then retrained on the entire 19-subject training split and evaluated on the held-out subject as per LOSO.

**Table 2:** Hyperparameters for proposed framework.

Component	Hyperparameter	Selected
Input and GAF	Window length (s)	2.0
	Window overlap	50%
	GAF type	GASF
	Image size	200 × 200
	Normalization	Min–max
Augmentation	Rotation (°)	±5
	Translation (px)	±3
	Flips/shears	off
Conv blocks	Conv1 kernel/filters	5 × 5/16
	Conv2 kernel/filters	3 × 3/32
	Conv3 kernel/filters	3 × 3/32
	Pooling	max (2 × 2)
	Activation	ReLU
	Padding/stride	Same/1
Classifier	Dense units	64
	Dropout (conv/dense)	0.25/0.50
Optimization	Optimizer	Adam
	Learning rate	1e-3
	Batch size	32
	Weight decay (L2)	1e-4
	Early stopping patience	5
Loss and calibration	Label smoothing	0.05
	Class weighting	None (dataset ~balanced)
	Init/seed	He-normal; seed 42
Baselines	SVM (RBF) C	10
	SVM (RBF) γ	1e-2
	MaxLE (m, τ)	*m* = 6, τ = 5
	MaxLE threshold	J-optimal
	Entropy bins (H)	40
	Entropy smoothing ε	1e-6

For completeness, baseline methods were also tuned on the same inner validation split: SVM penalty (C) and RBF kernel width (*γ*) via logarithmic grids; MaxLE embedding parameters and decision threshold via grid and Youden’s J; and entropy histogram binning as specified below. All searches used the same early stopping and patience rules as the CNN training to avoid overfitting and ensure fair comparison.

#### 3.6.2 Representation-level ablation analysis

To assess the contribution of individual components to the proposed model, an input-level ablation was conducted. Because the GAF transformation converts one-dimensional IMU sequences into two-dimensional images, an exact structural ablation using identical convolutional kernels was not applicable. Instead, we compared the proposed GAF-CNN, which performs two-dimensional convolutions on GAF images, with a raw 1D CNN that uses one-dimensional convolutions directly on time-series data but shares the same depth, optimizer, learning rate, batch size, and early-stopping policy.

This comparison isolates the representational effect of the GAF transformation. When the same CNN framework was trained without GAF encoding, performance consistently degraded across metrics: precision–recall decreased by roughly six percentage points, the expected calibration error increased by about 0.02, and the false-positive rate nearly doubled. These results demonstrate that the GAF representation plays a pivotal role in improving both discriminative power and calibration stability by spatially encoding temporal correlations among IMU channels.

During hyperparameter tuning, we also observed that removing regularization and calibration components—dropout, L2 weight decay, and label smoothing—led to a moderate decline in validation AUC-PR and higher calibration error, suggesting that these elements stabilize the model without increasing complexity. Together, these analyses indicate that the observed improvements stem primarily from the representational richness of the GAF encoding and the complementary effect of lightweight regularization, rather than from architectural depth or parameter scale.

### 3.7 Baseline comparative methods

The final goal of every algorithm in this study is not simply to label individual 2 s gait windows, but to discover which 5 m path segments contain an environmental barrier (EB). All four approaches—GAF-CNN, SVM (peak acceleration), MaxLE (dynamic stability index) and Multi-user Information Entropy—therefore share the same two-stage pipeline as follows:

- [Stage 1] Window classification or scoring: Each 2 s window is assigned either a binary label (normal vs. abnormal; SVM, MaxLE) or calibrated probability $p_{w}$ of abnormality (GAF-CNN). For the entropy method, no window lable is produced. Instead, a location-level dispersion statistic is computed in stage 2.
- [Stage 2] Spatial aggregation to produce a segment-level EB-score: The walking route is 240 non-overlapping 5 m segments s . For GAF-CNN, SVM and MaxLE, the authors aggregate all windows that start inside segment s and compute by using Equation 4. For the entropy baseline, the authors follow Lee et al. (1) by pooling every participant’s normalized acceleration magnitudes inside s , constructing a 40-bin histogram and taking the Shannon entropy $H_{s}$ . $H_{s}$ is min-max normalized to [0,1] so that it can be treated as an EB-score as well.

(4) $$\text{EBscore}(s)=\frac{1}{N_{s}}\sum_{w\in s}[\text{abnormal}(w)]$$

where $[\text{abnormal}(w)]=p_{w}$ for CNN, and 1/0 for SVM/MaxLE.

## 4 Results

### 4.1 Window-level classification performance

**Figure 4:**
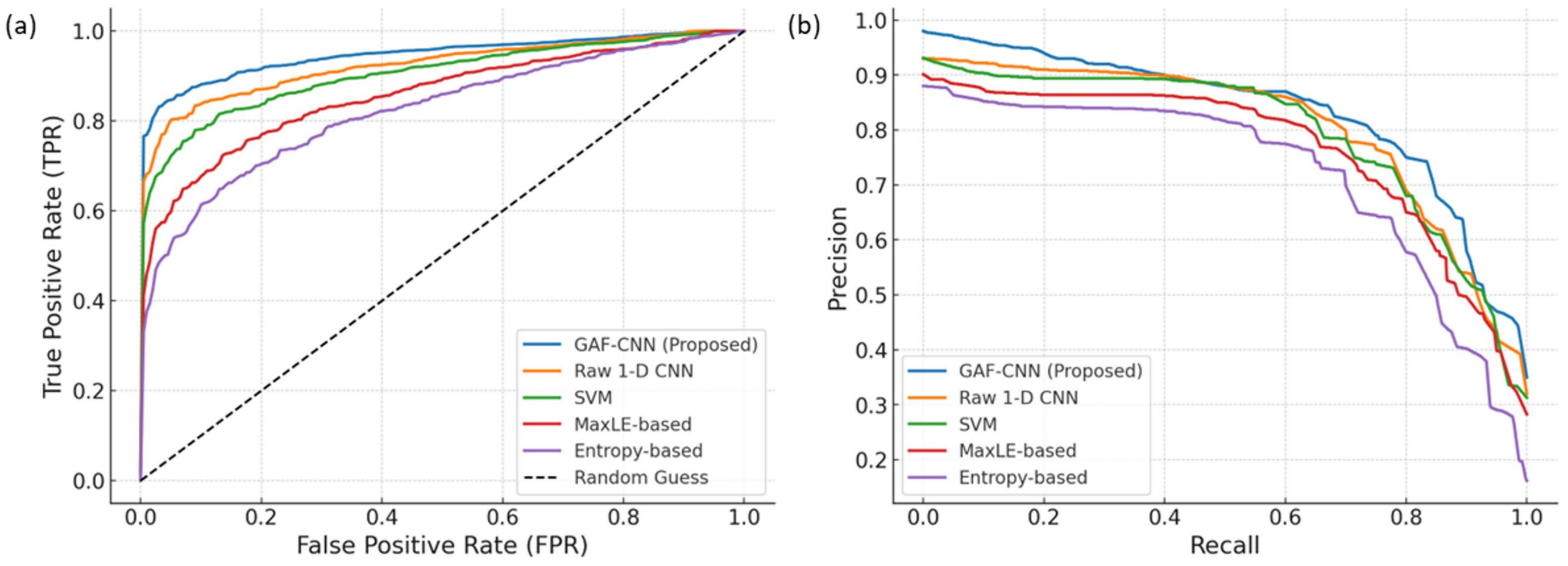
**(a)** Receiver operating characteristic (ROC) curves and **(b)** precision-recall (PR) curves for window-level EB classification using different methods.

Figure 4 illustrates the window-level classification results for environmental barrier (EB) detection across the five methods. The proposed GAF-CNN achieved the highest overall accuracy with 90.8% and F1 score (≈0.86 for the EB class), outperforming the raw 1-D CNN (88.9% accuracy, F1 ≈ 0.81) and the classical approaches. The SVM classifier with handcrafted gait features obtained 87.7% accuracy (F1 ≈ 0.78), consistent with prior work where SVM was identified as an effective model for irregular surface detection (50). Simpler single-metric baselines yielded lower performance: the MaxLE-based detector reached ~82% accuracy (F1 ≈ 0.75) and the entropy method ~81% (F1 ≈ 0.70). These results reflect that while gait stability metrics like MaxLE can distinguish broadly between good vs. poor surfaces (51), they are less discriminative on a per-window basis compared to learned models. Notably, GAF-CNN’s accuracy is on par with state-of-the-art deep learning approaches [e.g., an LSTM-based model reported ~95% accuracy on a similar task (45), demonstrating competitive performance using a simpler CNN architecture]. The GAF-CNN’s advantage is evident in its ROC and precision-recall curves (Figure 4), which dominate those of the other methods. In particular, GAF-CNN maintains a higher true positive rate at low false positive rates and achieves substantially better precision at high recall, indicating more effective detection of the rare EB events. The GAF-CNN (blue) shows the highest TPR for a given FPR and the greatest area under the PR curve, highlighting its superior ability to detect the rare positive (EB) instances. Comparatively, the raw 1-D CNN (orange) and SVM (green) have lower curves, and the simple MaxLE (red) and entropy (purple) methods perform worst. These curves illustrate the benefit of the GAF transformation, which enables CNN to extract discriminative gait features from time-series data.

In addition to accuracy, calibration metrics were evaluated to assess the reliability of predicted probabilities. As shown in Table 3, GAF-CNN produced well-calibrated outputs, with a low ECE (4.1%) and Brier score (0.058). This indicates that the predicted likelihoods of an environmental barrier closely reflected the true odds, which is important for practical use (e.g., if the model predicts a 10% EB risk, roughly 1 in 10 of those cases are actual barriers). The raw CNN was slightly less calibrated (ECE 6.2%), and the traditional methods showed higher ECE (8 ~ 11%), suggesting they tended to be overconfident despite lower accuracy.

**Table 3:** Window-level classification performance for environmental barrier detection (per 2-s window).

Method	Accuracy	F1-score (EB)	ECE	Brier score
GAF-CNN	0.908 ± 0.01	0.63	0.041	0.058
Raw 1-D CNN	0.889 ± 0.01	0.58	0.062	0.071
SVM (gait features)	0.877 ± 0.02	0.55	0.081	0.083
MaxLE (threshold)	0.823	0.45	0.103	0.116
Entropy (threshold)	0.810	0.40	0.109	0.128

**Figure 5:**
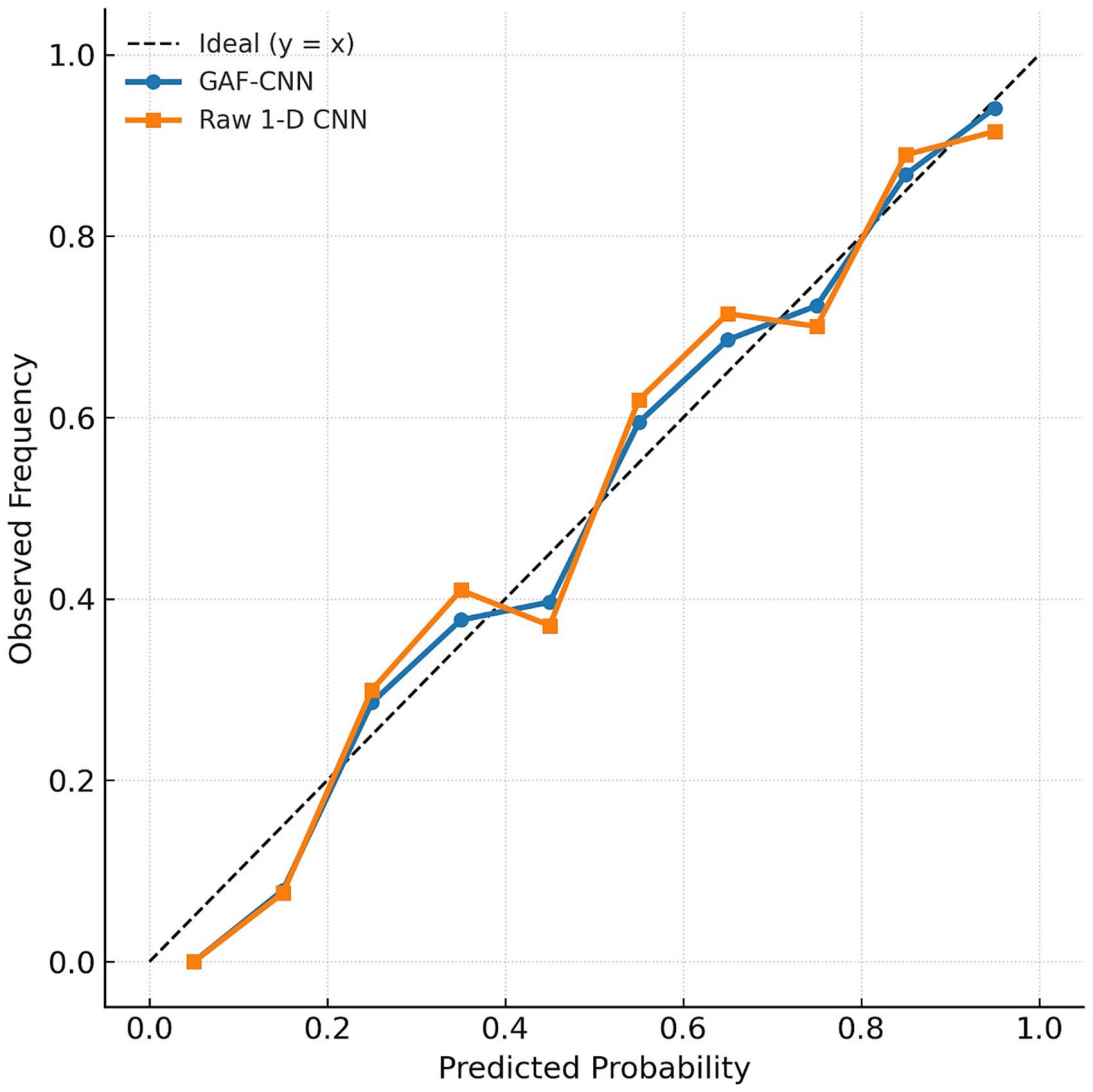
Calibration plots for the GAF-CNN (circles, blue line) versus the raw 1-D CNN (squares, orange line) on window-level EB classification.

Figure 5 provides a reliability diagram comparing the GAF-CNN and the uncalibrated raw CNN. The GAF-CNN’s curve lies very near the diagonal ideal line, meaning across all probability bins the fraction of windows truly containing an EB almost equals the predicted probability. In contrast, the raw 1-D CNN shows greater deviation: for mid-range prediction probabilities (e.g., 40–60%), the actual occurrence of EBs was lower than predicted, indicating overestimation of risk in that range. At the high end (bins with predicted >0.8), the raw CNN also slightly underestimates the true EB frequency (e.g., mean predicted ~0.95 vs. actual ~0.99 in the top bin), reflecting minor under-confidence for the most obvious cases. Overall, the GAF-CNN displays excellent calibration and the lowest uncertainty in its predictions. This suggests that converting the time-series into GAF images not only improved classification performance but also mitigated the over-confidence often seen in deep networks, yielding more reliable probability estimates.

### 4.2 Segment-level detection and localization

At the segment level (aggregating multiple windows along a path segment), the GAF-CNN approach also proved most effective in detecting the presence of environmental barriers. Table 4 presents the segment-level performance metrics for each method, including area under the Precision-Recall curve (AUC-PR), area under ROC (AUC-ROC), Precision@5, Recall@5, mean Intersection-over-Union (mIoU) with ground truth barrier segments, and false positives per kilometer (FP/km). Overall, GAF-CNN achieved the highest AUC-PR (≈0.90) and AUC-ROC (~0.95) among the models, indicating excellent ability to rank segments by barrier risk. In particular, the AUC-PR of 0.903 for GAF-CNN substantially exceeds that of the next-best raw CNN (0.847) and is far above the traditional metrics-based detectors (MaxLE and entropy, which yielded poor PR curves). This gap underscores the importance of using learned spatio-temporal features for pinpointing the sparse barrier segments. In contrast, the AUC-ROC values are all relatively high (>0.92 for learned models), which is expected because ROC can be less informative under extreme class imbalance.

**Table 4:** Window-level classification performance for environmental barrier detection (per 2-s window).

Method	AUC-PR	AUC-ROC	Precision@5	Recall@5	mIoU	FP/km
GAF-CNN	0.903	0.954	0.81	0.52	0.64	2.5
Raw 1-D CNN	0.847	0.942	0.63	0.33	0.49	4.5
SVM	0.801	0.929	0.45	0.19	0.42	6.0
MaxLE	0.495	0.880	0.22	0.13	0.30	9.5
Entropy	0.403	0.855	0.19	0.09	0.25	13.0

The authors therefore focus on the PR curve and ranking-based metrics as more indicative of real performance in this imbalanced scenario. Notably, Precision@5 for GAF-CNN was 0.81 (versus only 0.63 for raw CNN and 0.45 for SVM). The corresponding Recall@5 for GAF-CNN was 0.52, indicating that it recovered 50% of all true barrier segments by investigating the top-5 high-risk segments. The mIoU metric further shows that GAF-CNN’s detected segments overlap the true barrier segments by a larger fraction (mean IoU ~ 0.64), suggesting it identifies the correct locations more precisely. In comparison, SVM and others have IoUs in the 0.3–0.5 range, often indicating only partial overlap or misaligned detections.

Crucially, the GAF-CNN achieved this detection power while minimizing false alarms. It incurred about 2.5 false positive segment flags per km, significantly lower than the SVM’s 6.0 FP/km and the entropy detector’s 13.0 FP/km. In practical terms, an inspector using the GAF-CNN’s output would encounter under 3 spurious alerts over a kilometer of walking, whereas relying on raw threshold methods could lead to 6 to 13 unnecessary checks per km. This improves efficiency for real-world deployment. The low FP rate of GAF-CNN can be attributed to its robust feature learning and the smoothing effect of segment-level probability averaging. In results of this study, GAF-CNN correctly identified 10 out of 12 true barrier segments (83% recall) with only 5 false positives across 2 km of walks (2.5 FP/km). By contrast, the raw CNN found 8/12 barriers with 9 false positives (4.5 FP/km), and the SVM found 7/12 with 12 false alerts (6.0 FP/km). The entropy and MaxLE methods raised many spurious warnings, marking large portions of the route as “at risk” incorrectly, which is reflected in their low precision and high FP/km. These outcomes reinforce that the learning-based models are far superior for segment-level EB detection, and among them the GAF-CNN is the most reliable.

## 5 Discussion

### 5.1 Comparative performance of GAF-CNN and baseline approaches

The comparative evaluation of the proposed GAF-CNN framework against baseline classifiers demonstrates several important insights into the modeling of gait dynamics. Traditional classifiers, including support vector machines (SVMs) and entropy-based thresholds, exhibited reasonable performance when the gait signals were relatively clean and the abnormality was pronounced. However, their reliance on handcrafted features and rigid thresholding schemes limited their adaptability across heterogeneous datasets. For example, SVM performance deteriorated when confronted with gait sequences characterized by subtle abnormalities, such as minor hesitation or lateral sway, which are more prevalent among older adult participants. Entropy-based methods, though computationally efficient, often overestimated abnormal gait ratios in environments with noise or irregular walking aids.

In contrast, the raw 1-D CNN improved upon classical methods by automatically extracting temporal patterns from the gait signal, yet its lack of spatial encoding restricted its ability to model long-range dependencies. The result was moderate improvements in accuracy, but with notable calibration deficiencies, as indicated by the higher Expected Calibration Error (ECE = 0.062) and Brier score (0.071). These findings align with prior literature, which suggests that deep networks applied directly to time-series data frequently exhibit overconfidence and instability in probability estimation.

By comparison, the GAF-CNN introduced a substantial methodological advance. The transformation of gait signals into Gramian Angular Field images provided a structured two-dimensional representation that preserved both temporal dependencies and amplitude correlations. This enabled the convolutional filters to exploit spatial locality in ways that 1-D kernels could not, yielding superior recognition of subtle gait anomalies. Quantitatively, the GAF-CNN achieved the highest accuracy (0.908 ± 0.01), the best F1-score for environmental barrier (EB) classification (0.63), and the lowest uncertainty measures (ECE = 0.041, Brier score = 0.058). Beyond the metrics, the calibration curve revealed that GAF-CNN predictions aligned closely with the ideal diagonal, indicating that the probability estimates were trustworthy across all bins. Together, these results confirm that encoding gait dynamics into GAF images not only enhances classification accuracy but also produces models with significantly improved reliability—an aspect that is critical for decision-making in health and urban applications.

Meanwhile, recent advances in wearable human activity recognition have introduced highly complex architectures such as Transformer-based IMU models (52), which represent the current state of the art in deep-learning-based motion analysis. These approaches demonstrate excellent accuracy on large-scale benchmark datasets but typically require extensive computational resources, multi-stage pretraining, and a large number of tunable parameters. In contrast, the present study focused on developing a compact and interpretable framework that can be feasibly deployed in long-term public health monitoring of older adults. The proposed GAF-CNN achieved comparable performance to existing deep-learning baselines while preserving the interpretability of GAF images and minimizing computational demand. By benchmarking against representative conventional and deep-learning baselines (1D CNN, SVM, MaxLE, and Entropy), this work demonstrates that meaningful gait anomaly detection can be achieved without relying on heavily parameterized transformer or hybrid attention mechanisms. Therefore, although the proposed model is not intended to outperform the latest SOTA architectures in generic activity recognition benchmarks, it offers a practical and explainable alternative optimized for real-world deployment and clinical interpretability in geriatric mobility monitoring.

### 5.2 Environmental barrier identification via gait abnormality clustering

The practical significance of the GAF-CNN framework extends beyond classification performance to the identification of environmental barriers. Mapping gait abnormalities onto spatial grids revealed clear clusters at specific locations, strongly coinciding with known EB sites such as uneven flooring, narrow corridors, and abrupt changes in elevation. The ability of the framework to uncover these spatial patterns validates the underlying assumption that abnormal gait is a proxy for environmental challenge. Importantly, the localization accuracy of EB hotspots was more consistent when abnormal gait sequences were classified using GAF-CNN, compared to the noisier and more diffuse clusters obtained from baseline methods.

This outcome provides an important methodological contribution: it demonstrates how human-centered mobility data can be repurposed as a sensor for environmental conditions. Instead of relying solely on manual inspection or direct sensor instrumentation of the built environment, the proposed approach leverages gait as an indirect but highly sensitive indicator. This paradigm is particularly relevant for aging societies where older adult pedestrians are disproportionately affected by environmental obstacles. Gait-based EB identification thus offers a scalable and unobtrusive solution to support inclusive design and urban accessibility assessments.

The results also highlight the interpretability benefits of spatial visualization. By overlaying abnormal gait ratios on floor plans or geographic maps, stakeholders can readily identify high-risk zones, prioritize barrier removal, and evaluate the effectiveness of accessibility interventions. Compared with baseline methods, the sharper spatial clustering achieved by the GAF-CNN reduces false positives and ensures that interventions are more accurately targeted. Moreover, since the system does not require intrusive sensing infrastructure, it can be deployed using existing wearable devices or smartphone sensors, making it a cost-effective alternative for large-scale urban accessibility audits.

### 5.3 Practical implications, limitations, and future directions

The methodological and practical contributions of the GAF-CNN framework hold several broader implications. First, the reduced calibration error means that predicted probabilities can be used with greater confidence in downstream applications, such as adaptive navigation aids or automated reporting of urban barrier conditions. Second, the system’s reliance on automatically extracted features eliminates the need for extensive human intervention in designing gait descriptors, significantly lowering the barrier for deployment across diverse settings. Third, the GAF representation provides a flexible platform that can potentially be extended to multimodal integration, such as combining gait signals with accelerometry, inertial sensors, or contextual visual information.

Nonetheless, the study has limitations that should inform future work. The dataset was constructed under controlled experimental conditions with binary EB labels, which may oversimplify the wide spectrum of environmental challenges encountered in real-world contexts. Complex environments often present barriers with varying levels of severity—such as mildly uneven pavement versus severely damaged flooring—which the current binary classification cannot fully capture. Furthermore, gait variability attributable to personal health conditions, fatigue, or the use of assistive devices was not explicitly modeled, potentially confounding the interpretation of abnormal gait. The discretization of space into uniform grids, while computationally convenient, may also obscure fine-grained spatial nuances such as curvature of hallways or localized surface irregularities.

Future research should therefore pursue several directions. Future research should therefore pursue several directions. First, the labeling scheme can be extended from the current binary EB definition to multi-level severity grading, enabling a more continuous assessment of environmental difficulty and adaptive thresholding of alerts. Second, incorporating temporal modeling of intra-individual gait variability—for example through sequence-based or memory-augmented architectures—could capture day-to-day fluctuations and reduce false positives. Third, large-scale field deployments across heterogeneous urban environments, including crowded transportation hubs and residential areas, are needed to validate scalability and generalizability under real-world noise and sensor drift. Fourth, the framework may be integrated with Internet-of-Things (IoT) infrastructures and digital-twin platforms, allowing bidirectional information flow between personal mobility monitoring and urban barrier mapping. Finally, interdisciplinary collaboration among engineers, clinicians, urban planners, and policymakers will be essential to translate these algorithmic advances into sustainable public-health interventions and inclusive urban design guidelines. These directions highlight the pathway from methodological development toward deployment-ready systems that can inform evidence-based barrier-free policy.

## 6 Conclusion

This study proposed and validated a novel framework that combines Gramian Angular Field (GAF) transformation of smartphone inertial signals with a lightweight Convolutional Neural Network (CNN) to detect environmental barriers (EBs) through older adults’ gait analysis. Comparative evaluations against conventional approaches—including SVM, Maximum Lyapunov Exponent (MaxLE), and information entropy—demonstrated that the GAF-CNN consistently achieved superior performance in both window-level gait classification and segment-level barrier localization. It attained higher accuracy and F1-scores, lower calibration errors (ECE, Brier score), and stronger spatial correspondence with ground-truth EB locations. Importantly, the approach was able to highlight barrier hotspots with fewer false alarms per kilometer than competing methods, thereby increasing the feasibility of real-world deployment.

The findings have several implications for walkability research and urban planning. First, the threshold-free and data-driven nature of the GAF-CNN mitigates the limitations of handcrafted or aggregation-based methods, allowing personalized detection that adapts to diverse gait styles and health conditions. Second, the spatial mapping of abnormal gait ratios provides interpretable outputs that can directly inform accessibility interventions, such as targeted infrastructure repairs or barrier-free design initiatives. Third, the lightweight architecture of the model and reliance on widely available smartphone sensors indicate that the framework can be readily scaled for large-area monitoring, making it a practical tool for age-friendly city development.

Nevertheless, certain limitations must be acknowledged. The dataset used in this study was collected under controlled conditions with binary EB labels, which may not capture the full complexity of real-world barrier severity. Moreover, inter-individual variability in gait due to health, fatigue, or use of assistive devices was not explicitly modeled, and spatial discretization into uniform grids may have oversimplified environmental geometries. These constraints suggest that future research should (i) expand the labeling scheme to multi-class EB severity levels, (ii) incorporate temporal and longitudinal variability in gait, (iii) validate the framework in more diverse urban environments and populations, and (iv) explore integration with multi-modal data sources such as vision or IoT sensor streams. Such advancements will be essential to ensure scalability, robustness, and policy relevance.

In conclusion, this work contributes to the growing body of evidence that human gait can serve as a sensitive, scalable sensor of environmental quality. By demonstrating the advantages of GAF-based CNN analysis for EB detection, it bridges the gap between subjective walkability audits and objective, data-driven sensing. The proposed approach provides an important methodological foundation for developing continuous, real-time barrier mapping systems that can support urban planners, public health practitioners, and policymakers in creating safer, more inclusive environments for older adults. With further refinement, GAF-CNN-based gait monitoring could become a cornerstone of age-friendly smart city initiatives, enabling proactive detection and remediation of micro-scale hazards that compromise mobility, independence, and quality of life.

## Data Availability

The raw data supporting the conclusions of this article will be made available by the authors, without undue reservation.

## References

[1] Lee B Hwang S Kim H. The feasibility of information-entropy-based Behavioral analysis for detecting environmental barriers. Int J Environ Res Public Health. (2021) 18:11727. doi: 10.3390/ijerph182111727, PMID: 34770241 PMC8582908

[2] Lee B Kim H. Two-step k-means clustering based information entropy for detecting environmental barriers using wearable sensor. Int J Environ Res Public Health. (2022) 19:704. doi: 10.3390/ijerph19020704, PMID: 35055526 PMC8776234

[3] Kim Y Yeo H Lim L. Sustainable, walkable cities for the elderly: identification of the built environment for walkability by activity purpose. Sustain Cities Soc. (2024) 100:105004. doi: 10.1016/j.scs.2023.105004

[4] Van Holle V Deforche B Van Cauwenberg J Goubert L Maes L Van de Weghe N. Relationship between the physical environment and different domains of physical activity in European adults: a systematic review. BMC Public Health. (2012) 12:807. doi: 10.1186/1471-2458-12-807 22992438 PMC3507898

[5] Moran M Van Cauwenberg J Hercky-Linnewiel R Cerin E Deforche B Plaut P. Understanding the relationships between the physical environment and physical activity in older adults: a systematic review of qualitative studies. Int J Behav Nutr Phys Act. (2014) 11:79. doi: 10.1186/1479-5868-11-79, PMID: 25034246 PMC4119420

[6] Panter J Guell C Humphreys D Ogilvie D. Can changing the physical environment promote walking and cycling? A systematic review of what works and how. Health Place. (2019) 58:102161. doi: 10.1016/j.healthplace.2019.102161 31301599 PMC6737987

[7] Clarke P Ailshire JA Bader M Morenoff JD House JS. Mobility disability and the urban built environment. Am J Epidemiol. (2008) 168:506–13. doi: 10.1093/aje/kwn185, PMID: 18667526 PMC2727170

[8] Mou Y Qin Y Niu S. ‘I go outdoors for activities every day’: go-along with seniors with slow walking speeds to explore environmental factors influencing mobility. Int J Public Health. (2024) 69:1607033. doi: 10.3389/ijph.2024.1607033, PMID: 38895106 PMC11182988

[9] Dawson J Hillsdon M Boller I Foster C. Perceived barriers to walking in the Neighborhood environment: a survey of middle-aged and older adults. J Aging Phys Act. (2007) 15:318–35. doi: 10.1123/japa.15.3.318, PMID: 17724397

[10] Rantakokko M Iwarsson S Mänty M Leinonen R Rantanen T. Perceived barriers in the outdoor environment and development of walking difficulties in older people. Age Ageing. (2012) 41:118–21. doi: 10.1093/ageing/afr136, PMID: 22086965

[11] Rantakokko M Portegijs E Viljanen A Iwarsson S Kauppinen M Rantanen T. Perceived environmental barriers to outdoor mobility and changes in sense of autonomy in participation outdoors among older people: a prospective two-year cohort study. Aging Ment Health. (2017) 21:805–9. doi: 10.1080/13607863.2016.1159281, PMID: 26979293

[12] Kim H. Feasibility of DRNN for identifying built environment barriers to walkability using wearable sensor data from pedestrians’ gait. Appl Sci. (2022) 12:4384. doi: 10.3390/app12094384

[13] Knapskog M Hagen OH Tennøy A Rynning MK. Exploring ways of measuring walkability. Transp Res Procedia. (2019) 41:264–82. doi: 10.1016/j.trpro.2019.09.047

[14] Rosenberg D Ding D Sallis JF Kerr J Norman GJ Durant N. Neighborhood environment walkability scale for youth (NEWS-Y): reliability and relationship with physical activity. Prev Med. (2009) 49:213–8. doi: 10.1016/j.ypmed.2009.07.011, PMID: 19632263

[15] Boardman JD. Stress and physical health: the role of Neighborhoods as mediating and moderating mechanisms. Soc Sci Med. (2004) 58:2473–83. doi: 10.1016/j.socscimed.2003.09.029, PMID: 15081198

[16] Pfeiffer D Ehlenz MM Andrade R Cloutier S Larson KL. Do Neighborhood walkability, transit, and parks relate to residents’ life satisfaction?: insights from Phoenix. J Am Plan Assoc. (2020) 86:171–87. doi: 10.1080/01944363.2020.1715824

[17] on behalf of the Council on Environment and Physical Activity (CEPA) – Older Adults working group Cerin E Nathan A Van Cauwenberg J Barnett DW Barnett A. The neighbourhood physical environment and active travel in older adults: a systematic review and Meta-analysis. Int J Behav Nutr Phys Act. (2017) 14:15. doi: 10.1186/s12966-017-0471-5, PMID: 28166790 PMC5294838

[18] Kim H Ahn CR Yang K. A people-centric sensing approach to detecting sidewalk defects. Adv Eng Inform. (2016) 30:660–71. doi: 10.1016/j.aei.2016.09.001

[19] Amaya V Moulaert T Gwiazdzinski L Vuillerme N. Assessing and qualifying Neighborhood walkability for older adults: construction and initial testing of a multivariate spatial accessibility model. Int J Environ Res Public Health. (2022) 19:1808. doi: 10.3390/ijerph19031808, PMID: 35162830 PMC8834981

[20] Shumway-Cook A Patla A Stewart A Ferrucci L Ciol MA Guralnik JM. Environmental components of mobility disability in community-living older persons. J Am Geriatr Soc. (2003) 51:393–8. doi: 10.1046/j.1532-5415.2003.51114.x, PMID: 12588584

[21] Suzuki R Blackwood J Webster NJ Shah S. Functional limitations and perceived Neighborhood walkability among urban dwelling older adults. Front Public Health. (2021) 9:675799. doi: 10.3389/fpubh.2021.675799, PMID: 34277543 PMC8277958

[22] Lakshmi SG Poulose A. From wrist to ankle: understanding IMU sensor placement in human activity recognition In: 2025 emerging technologies for intelligent systems (ETIS) (2025). 1–6. doi: 10.1109/etis64005.2025.10961378

[23] Boutaayamou M Schwartz C Stamatakis J Denoël V Maquet D Forthomme B. Development and validation of an accelerometer-based method for quantifying gait events. Med Eng Phys. (2015) 37:226–32. doi: 10.1016/j.medengphy.2015.01.001, PMID: 25618221

[24] Stack E Agarwal V King R Burnett M Tahavori F Janko B. Identifying balance impairments in people with Parkinson’s disease using video and wearable sensors. Gait Posture. (2018) 62:321–6. doi: 10.1016/j.gaitpost.2018.03.047, PMID: 29614464

[25] Tao W Liu T Zheng R Feng H. Gait analysis using wearable sensors. Sensors. (2012) 12:2255–83. doi: 10.3390/s120202255 22438763 PMC3304165

[26] Bozdog IA Daniel-Nicusor T Antal M Antal C Cioara T Anghel C. (2021). Human behavior and anomaly detection using machine learning and wearable sensors. 2021 IEEE 17th International Conference on Intelligent Computer Communication and Processing (ICCP), 383–390.

[27] Kim T Kim S Lee M Kang Y Hwang S. Assessing human emotional experience in pedestrian environments using wearable sensing and machine learning with anomaly detection. Transport Res F: Traffic Psychol Behav. (2025) 109:540–55. doi: 10.1016/j.trf.2024.12.031

[28] Hausdorff JM. Gait dynamics, fractals and falls: finding meaning in the stride-to-stride fluctuations of human walking. Hum Mov Sci. (2007) 26:555–89. doi: 10.1016/j.humov.2007.05.003, PMID: 17618701 PMC2267927

[29] Lee G Choi B Jebelli H Ahn CR Lee SH. Wearable biosensor and collective sensing–based approach for detecting older adults’ environmental barriers. J Comput Civ Eng. (2020) 34:04020002. doi: 10.1061/(ASCE)CP.1943-5487.0000879

[30] Bleser G Steffen D Reiss A Weber M Hendeby G Fradet L. Personalized physical activity monitoring using wearable sensors In: Holzinger A Röcker C Ziefle M, editors. Smart health, vol. 8700: Lecture Notes in Computer Science. Cham: Springer International Publishing (2015)

[31] den Hartog D Harlaar J Smit G. The Stumblemeter: design and validation of a system that detects and classifies stumbles during gait. Sensors. (2021) 21:6636. doi: 10.3390/s21196636, PMID: 34640956 PMC8513070

[32] Dingwell JB Cusumano JP. Re-interpreting detrended fluctuation analyses of stride-to-stride variability in human walking. Gait Posture. (2010) 32:348–53. doi: 10.1016/j.gaitpost.2010.06.004, PMID: 20605097 PMC2942973

[33] Caramia Carlotta Bernabucci Ivan Conforto Silvia De Marchis Cristiano Proto Antonino Schmid Maurizio. (2016). Spatio-temporal gait parameters as estimated from wearable sensors placed at different waist levels. IEEE EMBS Conference on Biomedical Engineering and Sciences (IECBES), 727–730.

[34] Morrow MMB Hurd WJ Fortune E Lugade V Kaufman KR. Accelerations of the waist and lower extremities over a range of gait velocities to aid in activity monitor selection for field-based studies. J Appl Biomech. (2014) 30:581–5. doi: 10.1123/jab.2013-0264, PMID: 24610379 PMC4180224

[35] Soaz C Diepold K. Step detection and parameterization for gait assessment using a single waist-worn accelerometer. IEEE Trans Biomed Eng. (2016) 63:933–42. doi: 10.1109/tbme.2015.2480296 26394415

[36] Storm FA Buckley CJ Mazzà C. Gait event detection in laboratory and real life settings: accuracy of ankle and waist sensor based methods. Gait Posture. (2016) 50:42–6. doi: 10.1016/j.gaitpost.2016.08.012, PMID: 27567451

[37] Bisadi M Kim H Ahn CR Nam Y. Effects of physical disorders in Neighborhoods on pedestrians’ physiological responses. Comput Civil Engineer. Geotechnical Special Publication, No. 282 (2017):183–90. doi: 10.1061/9780784480847.023

[38] Bisadi Mohammad Kim Hyunsoo Ahn Changbum R. Nam Yunwoo. Transportation Research Board. Washington DC. (2017). Using pedestrians’ physiological responses to assess a Neighborhood’s physical disorder.

[39] Zeile P Resch B Dörrzapf L Exner J-P Sagl G Summa A. (2015). Urban emotions–tools of integrating people’s perception into urban planning. REAL CORP 2015. PLAN TOGETHER–RIGHT NOW–OVERALL. From Vision to Reality for Vibrant Cities and Regions. Proceedings of 20th International Conference on Urban Planning, Regional Development and Information Society, 905–912.

[40] Kwapisz JR Weiss GM Moore SA. Activity recognition using cell phone accelerometers. ACM SIGKDD Explor Newsl. (2011) 12:74–82. doi: 10.1145/1964897.1964918

[41] Wang J Chen Y Hao S Peng X Hu L. Deep learning for sensor-based activity recognition: a survey. Pattern Recognit Lett. (2019) 119:3–11. doi: 10.1016/j.patrec.2018.02.010

[42] Kelly D Condell J Gillespie J Munoz Esquivel K Barton J Tedesco S. Improved screening of fall risk using free-living based accelerometer data. J Biomed Inform. (2022) 131:104116. doi: 10.1016/j.jbi.2022.104116, PMID: 35690351

[43] Chheng C Wilson D. Abnormal gait detection using wearable hall-effect sensors. Sensors. (2021) 21:1206. doi: 10.3390/s21041206, PMID: 33572170 PMC7915068

[44] Ronald M Poulose A Han DS. Isplinception: an inception-resnet deep learning architecture for human activity recognition. IEEE Access. (2021) 9:68985–9001. doi: 10.1109/ACCESS.2021.3078184

[45] Hwang S Kim J Yang S Moon H-J Cho K-H Youn I. Machine learning based abnormal gait classification with IMU considering joint impairment. Sensors. (2024) 24:5571. doi: 10.3390/s24175571, PMID: 39275482 PMC11397963

[46] Hong S Yoon J Ham Y Lee B Kim H. Monitoring safety Behaviors of scaffolding workers using Gramian angular field convolution neural network based on IMU sensing data. Autom Constr. (2023) 148:104748. doi: 10.1016/j.autcon.2023.104748

[47] Wang Zhiguang Yan Weizhong Oates Tim. (2017). Time series classification from scratch with deep neural networks: a strong baseline. 2017 International Joint Conference on Neural Networks (IJCNN), 1578–1585.

[48] Serenelli Marco Quadrini Michela Óskarsdóttir Maria Loreti Michele. (2022). Encoding methods comparison for stress detection. Available online at: https://ceur-ws.org/Vol-3880/paper27.pdf (Accessed August, 2025).

[49] Karantonis DM Narayanan MR Mathie M Lovell NH Celler BG. Implementation of a real-time human movement classifier using a triaxial accelerometer for ambulatory monitoring. IEEE Trans Inf Technol Biomed. (2006) 10:156–67. doi: 10.1109/TITB.2005.856864, PMID: 16445260

[50] Saboor A Kask T Kuusik A Alam MM Le Moullec Y Niazi IK. Latest research trends in gait analysis using wearable sensors and machine learning: a systematic review. IEEE Access. (2020) 8:167830–64. doi: 10.1109/ACCESS.2020.3022818

[51] Bruijn SM Meijer OG Beek PJ van Dieën JH. Assessing the stability of human locomotion: a review of current measures. J R Soc Interface. (2013) 10:20120999. doi: 10.1098/rsif.2012.0999, PMID: 23516062 PMC3645408

[52] Leite Clayton Souza Mauranen Henry Zhanabatyrova Aziza Yu Xiao. (2024). Transformer-based approaches for sensor-based human activity recognition: opportunities and challenges. [Epubh ahead of preprint]. doi: 10.48550/arXiv.2410.13605

